# Challenges and Opportunities in Food Processing Using Innovative Techniques

**DOI:** 10.3390/molecules31162923

**Published:** 2026-08-21

**Authors:** Hanna Kowalska, Mariola Kozłowska

**Affiliations:** 1 Department of Food Engineering, Warsaw University of Life Sciences, 159c Nowoursynowska St., 02-776 Warsaw, Poland; 2 Department of Chemistry, Warsaw University of Life Sciences, 159c Nowoursynowska St., 02-776 Warsaw, Poland

## 1. Introduction

Alongside environmental concerns, the growing consumer demand for foods with high nutritional value, health-promoting properties, and sensory benefits has driven the development of innovative and sustainable food processing technologies. Non-thermal processing techniques, such as pulsed electric fields (PEFs), ultrasound (US), and cold plasma (CP), enhance mass transfer by modifying plant tissue, thereby improving processes such as drying and enrichment [1,2] and enabling hybrid methods [Contribution 1–2]. Enrichment during osmotic dehydration further increases the content of desirable compounds and reduces moisture content before drying [Contribution 2,11], while enriching bread with fermented additives, such as quinoa grains, and using quinoa malt can improve its nutritional value and sensory quality [Contribution 9]. Another important research direction involves the development of functional foods, as exemplified by dried kale-based snacks and high-fiber bars, in which blanching combined with various drying methods has been shown to affect the preservation of bioactive compounds, nutritional composition, and sensory qualities [Contribution 1]. Although these approaches offer considerable potential for improving the nutritional, functional, physicochemical, and sensory properties of food, their wider application requires further research to optimize processing conditions, enhance energy and resource efficiency, and ensure scalability and sustainability. The continued development and integration of these technologies may contribute to the production of innovative foods that combine high nutritional value, desirable sensory attributes, and reduced environmental impact.

The articles published in this Special Issue focus on several complementary topics, including innovative food processing technologies, the design of functional foods, the sustainable valorization of by-products, food safety and shelf life, and the relationships between food structure, physicochemical properties, nutritional value, and sensory qualities. These studies demonstrate how processing strategies, including pretreatment, advanced drying technologies, and fermentation, can be combined to preserve bioactive compounds, improve nutritional quality, and develop value-added foods with greater health-promoting potential.

## 2. Innovative Food Processing Technologies

The largest selection of articles is devoted to modern processing technologies, including the initial enrichment of NFC juices (not from concentrate) and the drying of carrots [Contribution 2], the use of hybrid drying [Contribution 1–2], the osmotic dehydration of beets [Contribution 11], the ultrasonic processing of fruit purees [Contribution 3], the lactic acid fermentation of oil plant by-products [Contribution 4], and the ultrasonic extraction of phenolic compounds [Contribution 7]. These studies demonstrate that the effectiveness of innovative technologies depends not only on the process parameters but also on the processing stage at which they are applied. Appropriate process design enables the modification of physicochemical, rheological, sensory, and functional properties, while minimizing the losses of bioactive compounds and improving process efficiency. A clear trend is the replacement of conventional methods with more energy-efficient technologies that are consistent with the principles of sustainable processing. Further progress requires a better understanding of the mechanisms underlying plant tissue transformation and the development of predictive models that integrate raw material properties, process parameters, and technological outcomes.

In the research included in this collection, the ultrasonic pretreatment of fruit purees increased process efficiency by 6.6% compared to a control group [Contribution 3], while cavitation improved compound extraction and modified the product structure. However, the effect of ultrasound on bioactive compounds remains incompletely understood, as it depends on processing conditions, matrix properties, and the presence of oxygen. Although cavitation-generated radicals can enhance the release of bioactive compounds, they can also accelerate the degradation of sensitive components such as anthocyanins and ascorbic acid. Under the conditions used in Contribution 3, no significant anthocyanin losses were observed, indicating that the processing parameters were appropriate.

Furthermore, enrichment with NFC juices [Contribution 2] and osmotic dehydration [Contribution 11] proved effective in modifying the nutritional and functional value of plant raw materials. Enriching carrots with chokeberry and cherry juices increased the polyphenol content by six to ten times, simultaneously improving sensory properties. The NFC juices acted as multicomponent impregnating solutions, introducing phenolic compounds, sugars, organic acids, pigments, and aromatic compounds that influenced antioxidant capacity, the drying process, and the product’s stability. Freeze-dried enriched carrots were characterized by glass transition temperatures in the range of 61.4–69.6 °C, indicating the high stability of these samples. Microwave–vacuum drying (MVD), a hybrid drying technique, produced carrots of comparable quality to freeze-drying while significantly reducing drying time. The high crispness of MVD products resulted from their porous microstructure, although they also exhibited greater hardness and fracture toughness, demonstrating that crispness is a complex property of dried products. Therefore, snack optimization should simultaneously consider porosity, hardness, bursting behavior, and sensory perception. A similar trend is supported by research on the hybrid convective–infrared–ultrasonic drying of kale leaves [Contribution 1], which demonstrated that integrating energy-delivery mechanisms with partial tissue destruction enables the production of products with high sensory and health-promoting qualities, with a better preservation of chlorophylls and carotenoids than freeze-drying. These results indicate the growing potential of hybrid technologies as effective and more accessible alternatives to traditional drying methods.

Traditional osmotic dehydration of beets in solutions of sucrose, inulin, xylitol, and erythritol with magnesium salts [Contribution 11] effectively increased magnesium content but reduced antioxidant activity. Therefore, using ingredients with naturally high antioxidant potential, such as fruit juices, herbs, and spices, seems more advantageous for developing nutritionally attractive snacks [3]. Because multicomponent osmotic solutions affect both mass transfer and product composition, their effects on the stability and bioavailability of bioactive compounds warrant further investigation.

In addition, lactic acid fermentation of oilseed by-products (cake) [Contribution 4] improved the functional properties of wheat bread, increased its bioactive compound and dietary fiber content, and supported the principles of a circular economy. However, replacing the flour with 9% fermented cake reduced the bread volume while increasing its hardness, gumminess, and stringiness. Future research should optimize flour substitution levels, bacterial strains, and fermentation conditions to maximize nutritional benefits while maintaining the desired technological and sensory quality.

In summary, the presented research indicates that the future development of innovative food processing will rely on integrating and optimizing modern unit operations tailored to specific food matrices, thereby enabling an effective control of mass transfer, the transformation of bioactive compounds, and product quality. Their industrial implementation will require pilot-scale validation, energy-efficiency assessment, an evaluation of by-product utilization, and comprehensive environmental analyses.

## 3. Design of Functional and Sustainable Foods with Emphasis on Safety and Shelf Life

An important research direction is the development of products with enhanced nutritional value using lesser-known plant raw materials. This field includes studies on gluten-free teff and soy flour blends [Contribution 5], quinoa sourdough starters enriched with quinoa malts [Contribution 9], and the characterization of mashua tuber flours [Contribution 10]. An appropriate selection of raw materials has been shown to increase the content of protein, fiber, minerals, and bioactive compounds, while also improving the technological and functional properties of the products. The addition of up to 10% quinoa malt improved fermentation, increased lactic acid content, broadened the volatile compound profile, and enhanced the nutritional value of quinoa sourdough starters [Contribution 9]. Black quinoa malt demonstrated particularly favorable properties, increasing protein content to 17.3%, fiber to 17.9%, and potassium and manganese, while the white variety was richer in magnesium, calcium, and copper. This indicates the need for further research into the effects of malting and fermentation on mineral bioavailability, as well as on the degradation of antinutritional compounds such as phytates. Mashua flours also showed great potential [Contribution 10], especially those of the black morphotype, which were characterized by a higher protein, fat, and iron content and greater thermal stability than the yellow morphotype.

The development of new raw materials is closely linked to the circular economy and the use of food industry by-products. Studies on the fermentation of oil plant by-products [Contribution 4] and the recovery of phenolic compounds from onion and grape processing by-products [Contribution 7] have confirmed their potential as a source of functional ingredients. Their extraction efficiencies depended on the type of raw material, solvent, and method used. The use of natural solvents and ultrasound-assisted extraction aligns with the principles of green chemistry and enables the design of processes focused on the recovery of specific groups of bioactive compounds. Further research should focus on developing efficient and scalable methods for the valorization of by-products and their use as sources of functional ingredients, in line with the growing importance of the circular economy and sustainable food design.

The potential of new raw materials should also be considered in the context of food safety and storage stability. Studies on the formation of nitrosamines in meat products [Contribution 6] and changes in the quality of mini kiwi during storage [Contribution 8] have demonstrated the importance of monitoring chemical and biochemical changes occurring during processing and storage. In meat products, the composition of the preservation system and simulated digestion conditions had a significant impact on nitrosamine formation, whereas, in mini kiwi, a controlled, modified atmosphere ensured an optimal preservation of firmness. Continuous ozonation supported selected antioxidant processes but caused minor skin damage, indicating the need for a further optimization of their storage methods.

These studies demonstrate that modern food production should incorporate new or lesser-known plant materials and by-products to enhance nutritional and health-promoting value. The development of such products should consider not only the content of bioactive compounds but also their bioavailability, sensory acceptability, and storage stability, as well as a minimization of undesirable compounds.

## 4. Relationships Between Stability, Physicochemical Properties, Microstructure, and Sensory Quality in Modern Food Design

Studies on dried carrots pre-enriched with NFC juices [Contribution 2] have demonstrated a close relationship between physicochemical properties and sensory acceptance. Water activity and glass transition temperature were found to be key indicators of product stability. Although freeze-drying resulted in lower water activity and greater stability of the glass transition state, microwave–vacuum drying (MVD) achieved a comparable quality, creating a fine-pore structure that promotes crispness and high sensory acceptance. MVD may therefore be an attractive alternative to freeze-drying, enabling the production of high-quality products with shorter processing times and, consequently, lower energy consumption.

Another important area of research is the rheological and textural properties of food, which determine the course of technological processes and product quality. The ultrasonic processing of berry purees was shown to affect the microstructure, viscosity, and stability of the product, while also promoting the preservation of bioactive compounds and sensory characteristics [Contribution 3]. The use of ultrasound increased process efficiency, apparent pulp viscosity, and pectin release, with minimal changes in anthocyanin and ascorbic acid content. Studies on gluten-free blends of brown teff (*Eragrostis* tef (Zucc.) *Trotter*) and soybean (*Glycine max* (L.) Merr.) flours [Contribution 5] have demonstrated that an appropriate selection of raw materials and particle size can enable the shaping of rheological properties by improving water binding and structural stability, among other factors, because of starch–protein interactions. Larger particle fractions increased water retention, while finer ones promoted hydration and swelling. The results confirmed the potential of these raw materials for developing high-protein, gluten-free products. Therefore, the comprehensive shaping of functional, rheological, and textural properties is crucial for designing foods with high technological, nutritional, and sensory quality.

The research published in this Special Issue demonstrates that designing the food of the future requires the simultaneous development of innovative processing technologies and the rational use of plant raw materials, by-products, and bioactive compounds in accordance with the principles of sustainable development. The results confirm the feasibility of developing high-nutrition-value products with desired technological and sensory qualities, while also indicating directions for further research, including industrial validation, the assessment of environmental performance, and implementation of the developed solutions.

We thank all the authors for preparing their high-quality articles, the reviewers for their rigorous and constructive comments, and the editorial team for their helpful support.

## Data Availability

No new data were created or analyzed in this study. Data sharing is not applicable to this article.
